# Cardiac vagal dysfunction moderates patterns of craving across the day in moderate to heavy consumers of alcohol

**DOI:** 10.1371/journal.pone.0200424

**Published:** 2018-07-17

**Authors:** Rhiannon E. Mayhugh, Paul J. Laurienti, Jason Fanning, Lise Gauvin, Keri J. Heilman, Stephen W. Porges, W. Jack Rejeski

**Affiliations:** 1 Laboratory for Complex Brain Networks, Wake Forest School of Medicine, Winston-Salem, North Carolina, United States of America; 2 Neuroscience Program, Wake Forest School of Medicine, Winston-Salem, North Carolina, United States of America; 3 Department of Radiology, Wake Forest School of Medicine, Winston-Salem, North Carolina, United States of America; 4 Translational Science Center, Wake Forest University, Winston-Salem, North Carolina, United States of America; 5 Department of Geriatric Medicine, Wake Forest University, Winston-Salem, North Carolina, United States of America; 6 Centre de recherche du Centre Hospitalier de l’Université de Montréal (CRCHUM) & Département de médecine sociale et préventive, École de Santé Publique, Université de Montréal, Montréal, QC, Canada; 7 Department of Psychiatry, University of North Carolina, Chapel Hill, North Carolina United States of America; 8 Department of Health and Exercise Science, Wake Forest University, Winston-Salem, North Carolina, United States of America; Mayo Clinic College of Medicine, UNITED STATES

## Abstract

**Background:**

Alcohol craving, a known correlate of vulnerability to Alcohol Use Disorder (AUD), has been found to be inversely related to cardiac vagal tone (CVT). Here we examine how resting CVT, CVT reactivity to a postural challenge, and their interaction influence craving during imposed alcohol abstinence and their usual drinking among moderate to heavy drinkers.

**Methods:**

Participants were recruited from the local community (final n = 29) and assessed for CVT functioning via respiratory sinus arrhythmia (RSA) at rest (RSA-rest) and during a postural challenge (RSA-react). Craving intensity was assessed throughout the day during 3-day periods of imposed alcohol abstinence (abstained days) and drinking as usual (normal days) via Ecological Momentary Assessment (EMA). Multilevel statistical modeling assessed relationships between patterns of CVT and diurnal craving. The primary hypothesis of interest was that the interaction of RSA-rest with RSA-react would be significantly associated with increased craving across the day.

**Results:**

Overall, craving increased throughout the day and significantly decreased after drinking (p < 0.001). There was a significant interaction between RSA-rest and RSA-react with plots revealing that this effect was driven by an aberrant craving pattern among participants with higher RSA-rest and a sluggish vagal brake in response to a postural shift—atypical RSA-react.

**Conclusion:**

Although additional research is needed to corroborate these findings, our results suggest that moderate-heavy drinkers characterized by higher RSA-rest and atypical RSA-react exhibit aberrant patterns of craving across the day that may represent a risk factor for AUD.

## Introduction

During the past decade of investigation into alcohol use disorders (AUDs) and the potential health benefits of moderate alcohol consumption, considerable attention has been devoted to the autonomic nervous system (ANS). The ANS is a component of the peripheral nervous system that provides the brain with sensory information from the body and, in turn, regulates bodily functions. When the two branches of this system, the sympathetic and parasympathetic, are working properly the body is able to maintain a healthy balance between appropriate reactions to environmental challenge and return to restorative, homeostatic states. This balance is thought to be affected by frequent alcohol consumption and play an important role in addiction [1].

Cardiac vagal tone (CVT) reflects the influence of the myelinated branch of the vagus nerve, which originates in the nucleus ambiguus, on the heart. CVT is commonly quantified by high frequency heart rate variability (HF-HRV) or respiratory sinus arrhythmia (RSA), and has been applied as a general index of ANS functional health [2]. Specifically, CVT captures the functional relationship between the heart and brainstem. In a healthy system, high CVT acts as a “vagal brake,” inhibiting the sympathetic nervous system’s influence on cardiac output. Consistent with Polyvagal Theory [2, 3], upon exposure to a stressor, CVT is reduced, lifting the “vagal brake”; thus enabling cardiac output and metabolic activity to increase to meet situational demands [4]. Dysfunctional CVT has implications for impaired psychological and physical health [5, 6].

CVT has been found to be sensitive to alcohol exposure and addiction with recent work suggesting that dysregulated ANS function may be an endophenotype for substance use disorders in general [7]. Recent systematic reviews have concluded that alcohol has a J-shaped effect on CVT, with moderate alcohol consumption increasing CVT and heavy consumption decreasing it [8, 9]. Altered CVT functioning has also been associated with craving. For instance, higher craving has been related to lower resting state CVT in alcohol dependence [10]. This altered CVT-craving relationship is important considering that higher levels of alcohol craving are thought to place one at increased risk for AUD [11]. For example, craving intensity across the day has predicted greater alcohol consumption in both dependent and non-dependent heavy alcohol consumers, as well as relapse in AUD recovery [12–14].

Whereas early investigations of CVT targeted resting state measures (RSA-rest) and led to the conclusion that higher RSA-rest is often found to be related to more flexible coping and more favorable regulation of emotional responses to stressful encounters, it was not long before researchers focused on reactivity during physical and psychosocial challenge (RSA-react; see reviews by [15, 16]. As specified by Polyvagal Theory [4], investigators have recognized the theoretical importance of the “braking function” of the myelinated vagal pathway as an important contribution to effective self-regulation. For example, research on tobacco has observed that smokers with blunted vagal reactivity to a psychological threat reported higher craving and were more prone to use smoking as a means of coping with stress than those with more normative responses to challenge [17].

At this junction we want to emphasize that with the exception of the work by Ashare and colleagues and a few other examples that we will discuss below, in the study of RSA-react, most research has focused on either how RSA-rest influences RSA-react to threat or challenge (see review by Balzarotti, Biassoni et al., 2017), or investigating RSA in response to appetitive cues [18–20]. Less attention has been given to studying whether different patterns of RSA-react to challenge might have prognostic value in understanding downstream emotional and/or behavioral responses. Moreover, although the current direction of research is to study RSA-rest and RSA-react as main effects, there is ample evidence that investigators should consider the interactive effects of these two metrics. First, there is strong evidence that the optimal phenotype for CVT is higher RSA-rest combined with vagal withdrawal under challenge, a typical RSA-react response [21–23]; however, data have shown that higher RSA-rest combined with a sluggish vagal brake in response to threat, an atypical RSA-react response, is a risk factor for both emotional [24] and behavior dysfunction [25]. Second, individuals with lower RSA-rest who manifest further withdrawal in RSA in response to challenge, a response in RSA-react that is atypical in light of the lack of vagal tone during resting state, have been shown to be at high risk for psychopathology [26], although Yaroslavsky and colleagues [24] found that augmentation of RSA to challenge is a risk factor for depression at any level of RSA-rest.

In a novel exploration into potential phenotypes of AUD vulnerability, the current study examined whether resting CVT (operationalized as RSA-rest), CVT reactivity measured as the change in RSA from a seated to standing posture (operationalized as RSA-react), or their interaction moderated alcohol craving across the day. Ecological Momentary Assessment (EMA) methodology [27] was used to assess craving during a 3-day period of normal patterns of drinking (normal days) and during 3-days of imposed alcohol abstinence (abstained days). On normal days, the impact of alcohol consumption on craving was also assessed. Given the substantial evidence for the relation between CVT and emotion [15] and that craving has been characterized as an emotional state [28], we hypothesized the presence of a significant interaction between RSA-rest and RSA-react with this effect potentially being driven by two phenotypes: higher RSA-rest and an atypical RSA-react response to challenge (a blunted vagal response) and/or lower RSA and an atypical response to challenge (higher levels of vagal withdrawal). The hypothesized interaction was tested using continuous variables, with exploratory plotting of interaction effects [29].

## Materials and methods

### Participants

Thirty-four moderate to heavy drinkers were recruited from the Winston-Salem area via study advertisements placed throughout the community, mass mailing, and business cards. The final sample included twenty-nine participants (13 men, 16 women) after excluding for missing data (n = 5). Enrollment criteria included those aged 24–60 years, alcohol consumption ≥ 50% of days in the past 3 months, average daily alcohol consumption of 1–3 drinks/day for women and 2–4 drinks/day for men for ≥ the past three years. Exclusion criteria included AUD diagnosis, binge drinking (NIAAA definition of ≥ 4 drinks for females, ≥ 5 drinks for males within 2 hours > once/month [30], consuming alcohol before noon > 3 times/month, active severe medical conditions, a score of > 20 on the Center for Epidemiological Studies Depression Scale (CES-D), history of neurological disease diagnosis, > 500 mg/day of caffeine, smoked > 1.5 packs/day, or positive urine drug test (methamphetamine, cocaine, marijuana, amphetamine, opiates, & benzodiazepines). Due to the association between body mass index (BMI) and blood-alcohol content (BAC), BMI was restricted to between 18.5 kg/m^2^ and 35 kg/m^2^ [31]. The Time Line Follow Back (TLFB), modified to record time of day, was administered during screening [32]. The Clinical Institute Withdrawal Assessment of Alcohol (CIWA-Ar) was used as a safety screening for alcohol withdrawal symptoms [33]. The Institutional Review Board of Wake Forest School of Medicine (IRB00028739) approved the study and written informed consent was obtained from all individual participants.

### Study overview

Both phone and in-person screens were conducted to ensure eligibility, complete informed consent, administer self-report questionnaires, and collect cardiac functioning data. The research visits occurred directly following the 3 consecutive normal and abstained days and included an fMRI scan and additional self-report questionnaires. The order of research visits was randomly assigned (3-day minimum wash out period). This report focuses on the RSA data collected during the screening visit and the EMA data collected throughout the 3 days prior to each research visit (normal and abstained days).

### Data processing for RSA calculation

During the screening visit, an electrocardiogram (ECG) was collected via a Biopac MP150 by Biopac Systems, Inc. (www.biopac.com, Goleta, CA). Three electrodes (one on each collar bone and one on the lower right rib for grounding) and a pulse oximeter (right index finger) were placed on the participant. The participant sat quietly for 5 minutes prior to data collection. Data were first collected in the seated position (5 minutes), then in standing position (2 minutes). Heart rate data were visually inspected and edited off-line with CardioEdit software (Brain-Body Center, University of Illinois at Chicago, 2007).

Heart rate and RSA were calculated from the ECG data using CardioBatch Plus software (Brain-Body Center for Psychophysiology and Bioengineering, University of North Carolina at Chapel Hill, 2016) consistent with procedures developed by [34]. CardioBatch Plus quantified the amplitude of RSA using age-specific parameters that are sensitive to the maturational shifts in the frequency of spontaneous breathing. The method includes the following steps: (1) timing sequential R-R intervals to the nearest millisecond; (2) producing time-base data by resampling the sequential R-R intervals into 500 msec intervals; (3) detrending the time-based series with a 21-point cubic moving polynomial that is stepped through the data to create a smoothed template then the template is subtracted from the original time-based series to generate a detrended residual series; (4) bandpass filtering the detrended time series to extract the variance in the heart period pattern associated with spontaneous breathing in adults (.12 - .40 Hz); and (5) transforming the variance estimates with a natural logarithm to normalize the distribution of RSA estimates [35]. These procedures are statistically equivalent to frequency domain methods (i.e., spectral analysis) for the calculation of the amplitude of RSA when heart period data are stationary [36]. RSA was quantified during each sequential 30-sec epoch and the averages within each condition were used in the data analyses. To calculate a RSA-react score, we subtracted the average 5-min RSA-rest value from the standing 2-min RSA value and then controlled for the 5-min RSA-rest values creating a residualized score.

### EMA protocol

Participants completed daily EMAs on study-issued iPhones. The iSurvey App (Harvest Your Data; Wellington, New Zealand) was utilized to assess craving intensity on an 11-point Likert scale, asking “Do you have a craving for alcohol right now?” The response scale was from 0 (“no craving”) to 10 (“extreme craving”). Likert assessments of stress and anxiety were also taken, but were not included in this analysis. To increase confidence that no alcohol was consumed during the abstained days, an alcohol content breath test was self-administered after each Likert survey. Breath tests were completed on Breathometer brand (www.breathometer.com, Burlingame, CA) testing devices and then BACtrack Mobile Pro (www.bactrack.com, San Francisco, CA,) for improved functionality.

EMA craving assessments were completed upon waking each morning, going to bed each evening, and when randomly prompted (total of 6 prompts sent between 9am & 9pm via the Audible Alerts application) on both normal and abstained days. The waking and going to bed assessments provided data points outside of the 9am-9pm window. During normal days, participants also recorded craving immediately prior to the initial drink and following the final drink of a drinking episode. These assessments established drinking commencement time and allowed for creation of dummy variables used to distinguish EMA reports occurring before and after drinking (see below). For each EMA assessment, participants recorded the “reason for completing the survey” (“I just woke up”, “I received a random alert”, “I am about to start drinking”, “I have just finished drinking”, or “I am going to sleep”). Upon enrollment, each participant received one-on-one training with the study coordinator (also available via text for assistance during the entire data collection period).

### EMA data processing and statistical analyses

The EMA reports resulting from random alerts, upon waking, or going to bed were stacked. A set of continuous and dummy variables were created to account for diurnal variations in cravings and to distinguish recordings occurring on abstained days versus normal days prior to and following the first drink. That is, the hour values of the timestamps of recordings were transformed into military time and the minute values were converted to fractions (e.g., 7:30 PM to 19.50, 9:45 PM to 21.75). Resulting times were centered at 15.00 (i.e., 3PM). EMA reports recorded after midnight but before going to bed were given values above 24:00 hours to accurately reflect the day on which they occurred (e.g., 25.25 for 1:15 AM, 26.00 for 2 AM, etc). In addition, EMA recordings occurring on abstained and normal days were stratified and then EMA recordings on normal days were further stratified as occurring either prior to (pre-drinking) or following (post-drinking) a drink with dummy variables. A first dummy variable contrasted EMA recordings occurring on normal days prior to the occurrence of the first drink (pre-drinking, value of 1) to all other occasions (value of 0). The second contrasted occasions on normal days after the occurrence of the first drink (post-drinking occasions, value of 1) to all other occasions. The reference category was therefore an abstained day.

Using SPSS, version 20, the distribution of craving reports was plotted and found to exhibit substantial positive skewness and could not be normalized using various mathematical transformations. To safeguard against erroneous conclusions from the raw data, the main analyses were replicated using other modeling approaches, for example, modeling for Poisson distribution. As determined by conducting Shapiro-Wilk tests, both RSA-rest and residualized RSA-react were normally distributed.

Since the data included repeated EMA reports of cravings that were nested within participants, the main statistical analysis strategy consisted of growth curve analyses, also known as multilevel modeling [37] and was performed with Hierarchical Linear and nonlinear Modeling software (HLM, version 7.0, Scientific Software International, Chicago, IL). First, a null multilevel regression model was applied to estimate the intraclass correlation (proportion of the total variance [including within day and between participant variance] attributable to between person variation) and to test whether or not the between participant variance in overall craving was statistically significant. Then, the variables capturing both linear and quadratic trends of time throughout the day were entered into the model along with the dummy variables contrasting EMA reports collected prior to and following drinking on normal days in comparison to abstained days. This allowed us to account for both diurnal variations in craving and the effects of drinking on craving in comparison to the 3-day period of abstinence. Finally, the centered values of the between participant variables of RSA-rest and residualized RSA-react, as well as their interaction, were entered into the model as continuous variables. Following recommendations of Aiken and West [29], significant interactions were further explored by plotting model-based estimates of craving as a function of four hypothetical cases: 1) RSA-rest set at 1 SD above the mean and residualized RSA-react set at 1 SD below than mean (higher RSA-rest/typical RSA-react); 2) RSA-rest set at 1 SD above the mean and residualized RSA-react set at 1 SD above than mean (higher RSA-rest/atypical RSA-react; 3) RSA-rest set at 1 SD below the mean and residualized RSA-react set at 1 SD above the mean (lower RSA-rest/typical RSA-react); and 4) RSA-rest set at 1 SD below the mean and residualized RSA-react set at 1 SD below than mean (lower RSA-rest/atypical RSA-react). A model was also run adjusting for age and sex.

## Results

### Daily patterns of craving

Among the 34 recruited participants, 2 did not comply with EMA prompting and 3 were missing RSA data. The remaining 29 participants provided a total of 1242 responses to the EMA survey with 650 occurring on abstained days, 462 before drinking, and 150 after drinking (592 total on normal days). Among the 1242 EMA reports, 170 were elicited upon waking in the morning, 130 because they were about to go to bed, and 922 because they received a random alert to complete an assessment. Individual participants provided a total of between 34 to 48 EMA responses combined across these categories (M = 42.8, SD = 3.5). EMA procedure compliance was above 80% and thus well above the 75% threshold expected for EMA studies. See Table 1 for participant characteristics. The mean and (SD) for RSA-rest was 5.76 (1.69), whereas it was -0.83 (0.72) for RSA-react (residualized RSA-react = -0.03 (0.69)).

**Table 1:** Sample characteristics: Mean (SD) or frequency.

Variable	Overall: EMA	Male (n = 13)	Female (n = 16)
Age	38.8 (10.9)	36.6 (6.6)	40.5 (13.3)
BMI	24.8 (3.8)	25.6 (3.5)	24.2 (4.0)
Race			
Black or African American	2	2	0
Asian	1	1	0
White	26	10	16
Alcohol Use			
Total Years Drinking	18.9 (10.8)	17.3 (7.2)	20.1 (13.1)
Years Maintained Current Drinking Pattern	8.3 (6.0)	9.2 (5.6)	7.6 (6.4)
Previous 3 Months (Time Line Follow Back)			
% of days that were drinking days	81.2 (16.0)	78.6 (16.4)	83.4 (15.8)
Average drinks consumed on drinking days	2.3 (0.73)	2.4 (0.26)	2.3 (0.96)

Males and females did not differ significantly in any of the above categories (alpha = 0.05).

**Fig 1:**
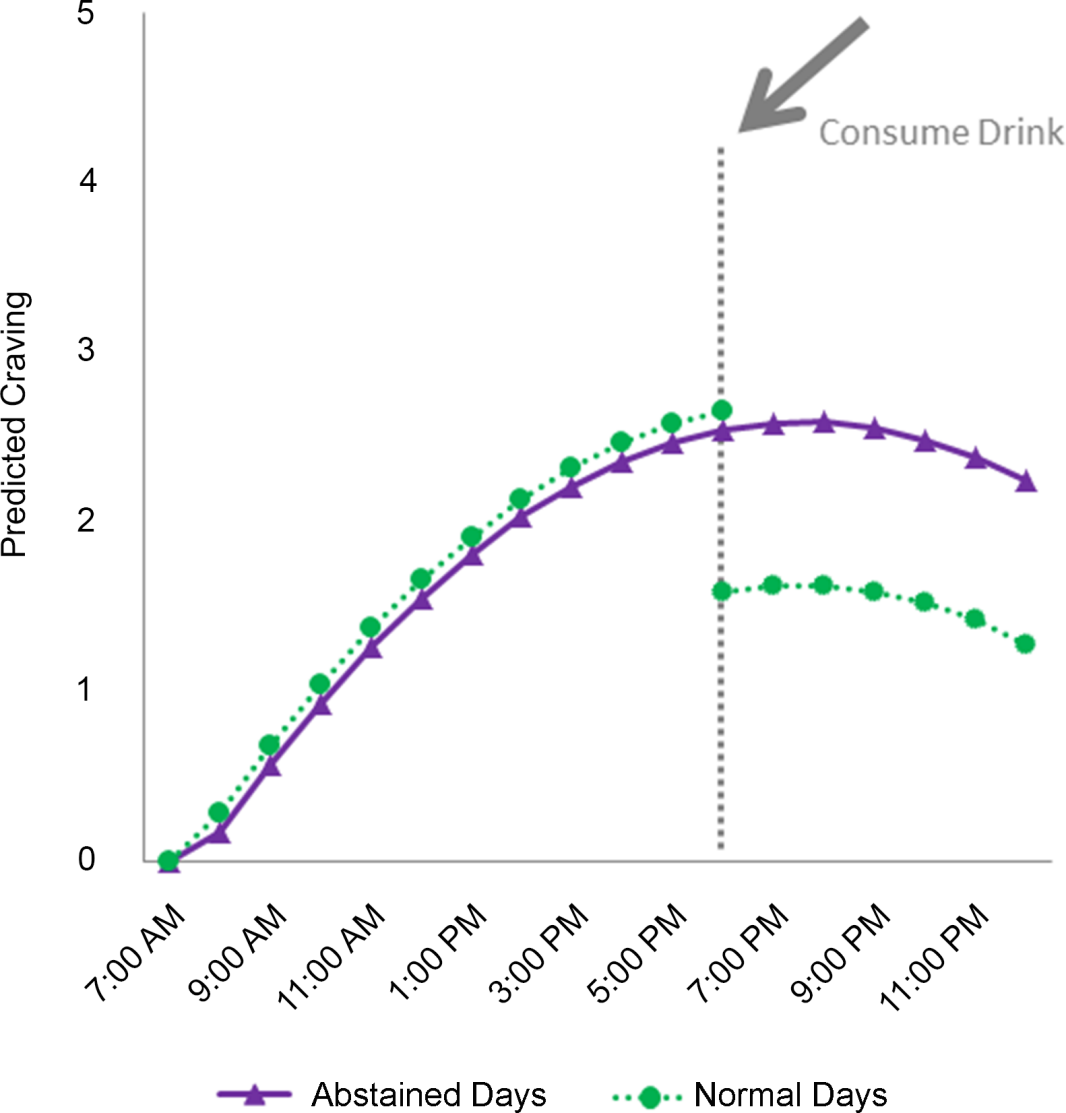
Estimated overall average effect across the 3-days of abstinence on craving responses by time of day and the effect of taking of drink on craving during the 3-day normal period.

In the first set of analyses, between participant variance in craving was statistically significant (X^2^(28) = 826.8, p<0.001). The intraclass correlation coefficient was 0.40 suggesting that most of the variance in craving was within person. Time effect modeling showed craving increased as the day progressed, but then dropped off later in the day depicting an inverted J-shape with linear and quadratic effects both statistically significant, p < 0.001. Further modeling revealed that craving was significantly lower following drinking on normal days (p < 0.001) in comparison to levels during abstained days. Craving was not different prior to drinking during the 3 normal days as compared to similar times during the 3 abstained days. Table 2 outlines results of this model and Fig 1 depicts predicted craving as a function of time of day and drinking using a drink consumed at 6 PM to illustrate the effect that drinking had on craving. Using alternative scaling for the craving variable with Poisson or dichotomized outcomes yielded identical results.

**Table 2:** Results of growth curve analyses examining the effects of time of day (linear & quadratic trends) and having a drink on reported craving for alcohol.

Effect in multilevel model	Unstandardized Coefficient (SE)
Overall level of craving	2.21 (0.32) ***
Linear effect of time of day (centered at 3PM)	0.17 (0.01) ***
Quadratic effect of time of day (centered at 3PM)	-0.02 (0.002) ***
Pre-Drinking (ref: abstaining)	0.11 (0.11)
Post-Drinking (ref: abstaining)	-0.96 (0.20) ***

* p < 0.05

** p < 0.01

*** p < 0.001

To determine the effects of CVT on craving, the influence of RSA-rest, residualized RSA-react, and their interaction on all effects of the previous model were assessed as continuous variables. Results (see Table 3) revealed that the linear and quadratic effects of time (diurnal patterns) and drinking effects were statistically significant (as in the first model). More importantly, the interaction of RSA-rest and residualized RSA-react significantly moderated the overall pattern of craving (p < 0.001) as well as the linear and quadratic effects of time (p values< 0.001).

**Table 3:** Results of growth curve analyses examining the moderating effects of RSA-rest, RSA-react, and their interaction on the effects of time of day (linear & quadratic trends) and having a drinking on reported craving for alcohol.

Effect in multilevel model	Unstandardized Coefficient (SE)	Unstandardized Coefficient (SE) adjusting for sex and age
Overall level of craving	2.33 (0.27) ***	2.20 (0.45) ***
Moderating effect RSA-rest	0.10 (0.19)	-0.08 (0.24)
Moderating effect of RSA-react	1.03 (0.48) *	0.80 (0.54)
Moderating effect of interaction of RSA-rest & RSA-react	1.08 (0.23) ***	0.94 (0.26) **
Moderating effect of female sex	-	0.25 (0.62)
Moderating effect of age	-	-0.04 (0.03)
Linear Effect of time of day (centered at 3PM)	0.16 (0.01) ***	0.21 (0.02) ***
Moderating effect RSA-rest	0.05 (0.01) ***	0.05 (0.01) ***
Moderating effect of RSA-react	0.07 (0.02) ***	0.09 (0.02) ***
Moderating effect of interaction of RSA-rest & RSA-react	0.04 (0.01) ***	0.05 (0.01) ***
Moderating effect of female sex	-	-0.08 (0.02) ***
Moderating effect of age	-	0.0008 (0.001)
Quadratic effect of time of day (centered at 3PM)	-0.02 (0.002) ***	-0.02 (0.003) ***
Moderating effect RSA-rest	0.002 (0.001)	0.005 (0.002) ***
Moderating effect of RSA-react	-0.009 (0.004) *	-0.007 (0.004)
Moderating effect of interaction of RSA-rest & RSA-react	-0.014 (0.002) ***	-0.011 (0.002) ***
Moderating effect of female sex	-	0.004 (0.005)
Moderating effect of age	-	0.0006 (0.0002) *
Pre-drinking (ref: abstaining)	0.09 (0.11)	0.27 (0.17)
Moderating effect RSA-rest	0.11 (0.08)	0.16 (0.09)
Moderating effect of RSA-react	0.15 (0.19)	0.28 (0.21)
Moderating effect of interaction of RSA-rest & RSA-react	0.10 (0.10)	0.15 (0.11)
Moderating effect of female sex	-	-0.32 (0.23)
Moderating effect of age	-	0.01 (0.01)
Post-drinking (ref: abstaining)	-0.97 (0.19) ***	-1.37 (0.34) ***
Moderating effect RSA-rest	-0.26 (0.12) *	-0.21 (0.16) *
Moderating effect of RSA-react	-0.44 (0.32)	-0.64 (0.37)
Moderating effect of interaction of RSA-rest & RSA-react	0.04 (0.14)	-0.08 (0.17)
Moderating effect of female sex	-	0.67 (0.45)
Moderating effect of age	-	0.01 (0.02)

* p < 0.05

** p < 0.01

*** p < 0.001

**Fig 2:**
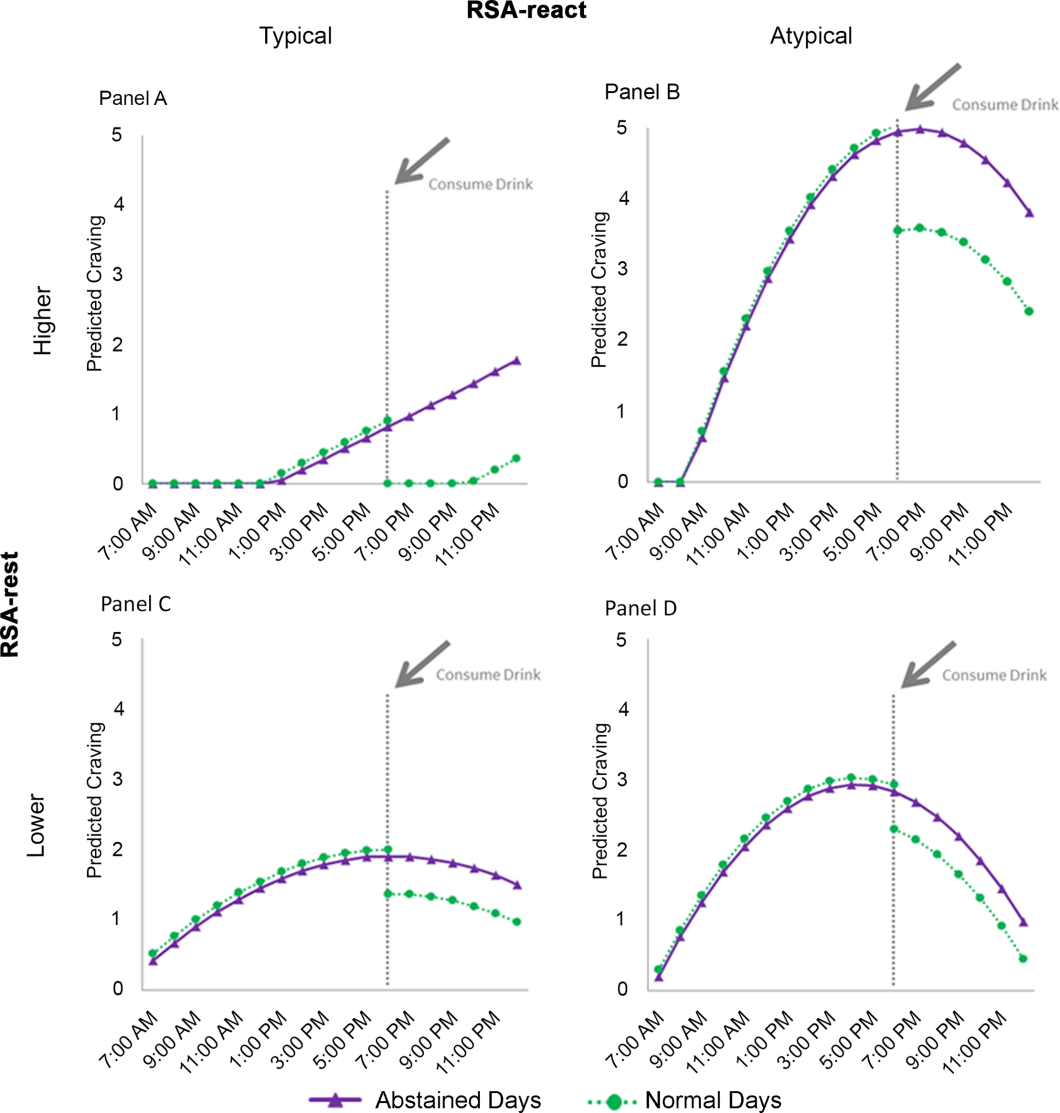
Plot of model-based estimates of craving across the 3-days of abstinence and before and after taking a drink during the 3-day normal period for four hypothetical cases. 1) RSA-rest set at 1 SD above the mean and residualized RSA-react set at 1 SD below than mean (higher RSA-rest/typical residualized RSA-react; Panel A); 2) RSA-rest set at 1 SD above the mean and residualized RSA-react set at 1 SD above than mean (higher RSA-rest/atypical residualized RSA-react; Panel B); 3) RSA-rest set at 1 SD below the mean and residualized RSA-react set at 1 SD above the mean (lower RSA-rest/typical residualized RSA-react, Panel C); and 4) RSA-rest set at 1 SD below the mean and residualized RSA-react set at 1 SD below than mean (lower RSA-rest/atypical residualized RSA-react; Panel D).

To more clearly depict the main effects and their interaction, we plotted the model-based estimates for four hypothetical RSA phenotypes: 1) higher RSA-rest/typical RSA-react; 2) higher RSA-rest/atypical RSA-react; 3) lower RSA-rest/typical RSA-react); and 4) lower RSA-rest/atypical RSA-react. Results showed that individuals with a higher RSA-rest and atypical RSA-react phenotype had a very unique pattern in their craving responses. That is, as shown in Fig 2, Panel B, they had a steeper increase in craving as the day progressed in comparison to those with a higher RSA-rest/typical RSA-react score (Panel A). The two other phenotypes, those with lower RSA-rest and either typical or atypical RSA-react showed both moderate increases in craving throughout the day and decreases in craving upon taking a drink. In our data set, older age was associated with lower RSA-rest (r = -0.50, p<0.006) and women had lower RSA-rest than men (M = 5.56, SD = 2.13 vs. M = 6.00, SD = 0.93), but there were no associations with residualized RSA-react. These values closely match RSA values observed over two decades ago by Byrne et al. among a sample of adults ranging in age between 20 and 87 years [38]. To examine possible confounding of the effects of RSA-rest, residualized RSA-react, and their interaction by age and sex, we reran the analyses while adjusting for age and sex. As shown in Table 3, the interaction remained statistically significant despite controlling for age and sex.

## Discussion

As shown in Fig 1, craving responses for this sample of moderate to heavy drinkers across the day during a 3-day period in which alcohol was consumed as usual and during a 3-day period of imposed abstinence were modest with a peak average value of just below 3 on a visual analogue scale that ranged from 0–10. Yet, even at these levels, it was clear that drinking effectively reduced cravings during days of normal consumption. Moreover, consistent with our primary hypothesis, there were important between-group differences based on the statistically significant interaction effect between RSA-rest and RSA-react.

As supported by the data in Table 3 and the plots in Fig 2, the driving influence behind the significant interaction term was the aberrant craving pattern of individuals with a phenotype characterized by higher RSA-rest in combination with an atypical RSA-react response (Fig 2, Panel B). Specifically, a case with an RSA-rest score 1SD above the mean and a RSA-react score 1SD above the mean—a sluggish vagal brake in response in a postural challenge—had a dramatic increase in craving as the day progressed. This effect becomes most apparent when contrasting the craving pattern of this phenotype with that of someone with a higher RSA-rest and a typical vagal withdrawal to challenge (Fig 2, Panel A), reinforcing the adaptive role that this latter phenotype has on emotional and behavioral functioning [21–23]. The aberrant pattern of craving observed for those with a higher RSA-rest and atypical RSA-react response is consistent with data published by Heilman and colleagues [25] concerning the dysfunction social behavior of children with selective mutism and data demonstrating that this phenotype is at an increased risk for depression (Yaroslavsky et la., 2013).

These data are particularly interesting considering that high RSA-rest is often associated with enhanced self-regulation and better psychological health [4, 6]. In fact, in the alcohol literature, craving has been reported to be inversely related to RSA assessed during a resting state [10]. What the current data suggest is that, although main effects for RSA-rest do occur, the conceptual significance of RSA-rest values need to be interpreted in light of RSA-react. Considering that craving has been characterized as an emotional state [28], individuals with higher RSA-rest/atypical RSA-react may be experiencing more intense craving across the day in a manner consistent with the flexibility hypothesis proposed by Balzarotti and colleagues [15]; that is, they have a propensity to experience more intense emotional responses—positive or negative. Although lower RSA-rest (typical or atypical RSA-react) was not associated with elevated cravings for alcohol, there was a continuum of craving with changes in RSA-rest and RSA-react. As shown in Fig 2, higher RSA-rest/typical RSA-react had the lowest pre-drink craving. Craving then increases as the CVT profiles move from lower RSA-rest/typical RSA-react, lower RSA-rest/atypical RSA-react, and then to higher RSA-rest/atypical RSA-react. These results also do not rule out the possibility that those with lower RSA-rest may well be using drinking to counter dysphoric mood [39]. This hypothesis deserves to be tested in future research [15].

We did not have a sufficient numbers of participants in various subgroupings of RSA-rest and RSA-react to explore how changes in RSA during challenge within various subgroups may be related to change in heart rate responses. Such analyses are warranted in future research since Porges and colleagues have observed that an uncoupling of change in RSA with change in heart rate during periods of challenge is consistently related to dysfunctional social behavior [4, 40–42] and may well be important in understanding alcohol craving. Needless to say, the patterns observed in the EMA craving responses for a phenotype with higher RSA-rest and an atypical RSA-react to postural challenge are disconcerting, and perhaps highlights one pathway by which craving is linked to AUD [11, 12, 14].

Due to our exclusionary criteria for those diagnosed with AUD or alcohol dependence, the drinking behavior of moderate to heavy drinkers is likely predominately goal-directed reward seeking (e.g., a means to increased social connection) and not driven by automatic processes. Goal-directed reward seeking occurs when the decision to consume is driven by a conscious evaluation of the hedonic value of alcohol outweighing competing priorities, such as negative consequences related to use [43, 44]. This is in contrast to automatic drinking behavior seen in addiction that is unconscious, stimulus dependent, and driven by dopaminergic reward pathways [45]. Considering the relationship between craving and abnormal CVT functioning [8, 10], the higher overall craving responses observed in the moderate to heavy drinkers with a higher RSA-rest/atypical RSA-react phenotype suggests that they may represent a subpopulation with greater vulnerability to drinking escalation and future AUD. As the craving response of this phenotype may be due to the higher hedonic value of alcohol in this subgroup, likely involving unconscious processes, a hypothesis exploring functional brain network studies may provide further insight. Within this theoretical framework, those with higher hedonic value of alcohol would be more likely to prioritize drinking over competing interests or avoidance of negative consequences related to drinking, again putting them at greater risk for AUD.

A final point that we would like to mention is the complexity underlying the concept of RSA reactivity. CVT functioning, specifically the myelinated vagal pathways originating in nucleus ambiguus, has shown to be important in self-regulation; however, emotional and/or behavioral responses can be either approach or avoidance related [26]. This distinction is important to consider as a number of papers have demonstrated relationships between cue reactivity, HRV, and craving [18, 19]. We infer from these HRV studies, since there are strong correlations between overall heart rate variability and RSA, although quantifying RSA provides a metric that is more tightly linked to neural mechanism [46]. For example, Garland and colleagues [47] found higher HRV reactivity with greater attentional bias to drinking-related cues in alcohol-dependent individuals. Specifically, relapsing alcohol-dependent patients had higher HF-HRV reactivity to stress-primed alcohol cues. Within the current study design, we intentionally chose to examine patterns of RSA-react to a postural challenge—transitioning from a seated to standing position—because a postural shift elicits a reflex and reflexes should be minimally influenced by contextual and other psychological variables. We then used this information to characterize how aberrant tonic and phasic RSA responses may interact to create physiological phenotypes that might explain downstream differences in craving. This approach parallels some extant work by Porges and colleagues (e.g., Heilman et al., 2012).

Future research could expand upon these findings by utilizing portable technology to record ambulatory assessments of RSA throughout the day. These data would be ideal for evaluating the synchrony between RSA and EMA measurements of subjective craving and stress. There is considerable research supporting the relationship between CVT and negative emotion [15]. This line of research would provide insight to a wide range of stressors that are meaningful to individual participants and their corresponding, real time, CVT and craving responses. Since we have shown that moderate to heavy drinkers who reported higher levels of craving also experienced greater stress across the day and were more likely to experience higher craving when experiencing higher levels of stress [48], future research may find that these individuals also have suboptimal CVT functioning during periods of high stress. A suboptimal autonomic response to daily stress could add important insight to potential vulnerability to addiction within this group as the link between stress, craving, and addiction has been well established [49–52].

Although this study has a number of strengths it is not without limitations. First, although we did not observe that sex has a significant impact on the results of this study, in fairness, we were not powered to detect such effects and future work should examine this possibility. Future work should also examine age as a possible moderating variable since previous work has reported decreases in heart rate variability with age. For example, Byrne and colleagues demonstrated age-related declines in HRV in a healthy population, absent of major health issue know to affect HRV such as substance abuse [38]”.

Second, differences in craving *during* drinking have been related to amount consumed and differ between dependent and non-dependent drinkers [53, 54]. This study only assessed craving after their final drink (upon going to bed) which minimized disruption of participants typical drinking, reducing chances of the craving assessment itself influencing ratings. And third, the random alerts only occurred between 9am– 9pm to avoid disruptions in sleep. On some occasions, this may have led to a lapse in data sampling. However, the participant-triggered alerts (i.e. “I am going to sleep”) provided additional data outside the random alert schedule.

In summary, to our knowledge this is the first investigation in the alcohol literature to examine how abnormal CVT reactivity influences alcohol craving both during periods of drinking as usual and abstinence in moderate to heavy drinkers. Individuals with a higher RSA-rest in combination with an atypical RSA-react phenotype were observed to exhibit higher cravings across the days than their peers with different phenotypes. In fact, the data suggested that unlike their peers, individuals with a higher RSA-rest and atypical RSA-react phenotype experienced ongoing and substantial increases in cravings throughout the day. Future research is needed to replicate these findings and to investigate whether the self-regulatory dysfunction of CVT in persons who are moderate to heavy drinkers places them at increased risk for AUD.
